# Cecum perforation associated with a calcium polystyrene sulfonate bezoar - a rare entity

**DOI:** 10.1590/2175-8239-JBN-2018-0158

**Published:** 2018-12-06

**Authors:** David Carvalho Fiel, Iolanda Santos, Joana Eugénio Santos, Rita Vicente, Susana Ribeiro, Artur Silva, Beatriz Malvar, Carlos Pires

**Affiliations:** 1 Hospital do Espírito Santo de Évora Évora Portugal Hospital do Espírito Santo de Évora, Largo Senhor da Pobreza, Évora, Portugal.

**Keywords:** Hyperkalemia, Potassium, Calcium, Bezoars

## Abstract

Hyperkalemia is one of the most common electrolyte disorders, responsible for a high number of adverse outcomes, including life-threatening arrhythmias. Potassium binders are largely prescribed drugs used for hyperkalemia treatment but unfortunately, there are many adverse events associated with its use, mostly gastrointestinal. Identification of patients at highest risk for the serious complications associated with the current potassium binders, such as colon necrosis and perforation, could prevent fatal outcomes. The authors present a case of a 56-year-old man with secondary diabetes and chronic renal disease that was treated for hyperkalemia with Calcium Polystyrene Sulfonate (CPS). He later presented with acute abdomen due to cecum perforation and underwent ileocecal resection but ultimately died from septic shock a week later. During surgery, a solid white mass was isolated in the lumen of the colon. The mass was identified as a CPS bezoar, a rare drug-mass formed in the gastrointestinal tract that contributed to the perforation. A previous history of partial gastrectomy and vagothomy was identified as a probable risk factor for the CPS bezoar development. Hopefully, the two new potassium binders patiromer and (ZS-9) Sodium Zirconium Cyclosilicate will help treat such high-risk patients, in the near future.

## Introduction

Hyperkalemia is one of the most common electrolyte disorders, responsible for a high number of adverse outcomes, including life-threatening arrhythmias^1^. Although little is known about the true incidence and prevalence of hyperkalemia in the general population due to the lack of epidemiological studies, it can affect up to 50% amongst the highest risk patients: those with chronic kidney disease (CKD) and end-stage renal disease on dialysis (ESRD)^1^. A correlation has also been established between hyperkalemia and other risk factors such as older age, Caucasian race, diabetes mellitus (DM) and renin-angiotensin-aldosterone system inhibitors (RAASi) use^1^. Treating such patients can be rather challenging, as they are the ones that benefit the most from the inhibition of the renin-angiotensin-aldosterone system but also are the most at risk of life-threatening hyperkalemia. As shown in various retrospective and observational studies, several patients who should be medicated with RAASi according to guidelines have been prescribed with lower-than-therapeutic doses or have discontinued this medication due to hyperkalemia, with consequent worse outcomes and mortality^2^. This emphasizes the importance of strategies that can lower serum potassium levels and maintain levels in the normal range, such as potassium binders (PB). PB are artificial resins that bind potassium ions in the gastrointestinal tract (GIT), exchanging these ions for calcium (Ca^2+^) or sodium (Na^+^) and hydrogen (H^+^) cations, therefore preventing potassium from being absorbed.

There are two classes of PB widely commercialized in most countries: calcium polystyrene sulfonate (CPS) and sodium polystyrene sulfonate (SPS), differing in the cation attached to the resin that is exchanged with potassium in the intestinal lumen. However, these drugs have poor digestive tolerability and cause adverse events, which commonly lead to the discontinuation of the drug by patients themselves: constipation, diarrhea, and abdominal pain.

Patiromer and ZS-9 (sodium zirconium cyclosilicate) are new PB not yet available in some countries, with good tolerability and promising results regarding the treatment of patients with hyperkalemia^3^.

In this article, the authors describe a case of a severe adverse event associated with PB, namely a cecum perforation associated with a PB bezoar (Table 1).

**Table 1:** Main clinical events of the presented case

	PRESENTATION	22^nd^ DAY OF ADMITTANCE	25^th^-30^th^ DAY OF ADMITTANCE	33^rd^ DAY OF ADMITTANCE
Observation	Right foot ulcer and cellulitis.	Hyperkalemia (K+ 6.0 mmol/L).	Hypokalemia (K+ 2 mmol/L).	Cecum perforation with peritonitis.
Management	• Long-term Piperacilin/Tazobactan iv. • Amputation of the 2^nd^-3^rd^ right toes.	• Dietary potassium restriction. • Insulin dose increase. • CPS prescription.	• CPS suspension. • Spironolactone prescription. • Large amounts of K^+^ iv.	• Ileocecal resection (bezoar removed). •Imipenem/Cilastatin initiation.
Outcome	Amputation of the right leg.	Hypokalemia (2,0 mmol/L).	Refractory Hypokalemia. CPS bezoar formation	Death due to septic shock.

## Case presentation

A 56-year-old Caucasian man presented to the Emergency Department (ER) with a two-month-lasting painful lesion in his right foot. The patient had a history of chronic alcoholic pancreatitis and secondary DM at young age, which later culminated in diabetic kidney disease. At hospital admission, he had stage 4 CKD with renal tubular acidosis type 4 (ATR 4). Other significant conditions were hypertension, history of duodenal ulcer with stenosis resolved by partial gastrectomy (with Bilroth II and vagothomy) at the age of 45, ischemic stroke at the age of 52, hypothyroidism, and major depressive disorder. He was chronically medicated with insulin, enalapril, nifedipine, darbapoetin alfa, sodium bicarbonate, clopidogrel, rosuvastatin, levothyroxine, escitalopram, and pantoprazole. He had also been medicated with a PB (calcium polystyrene sulfonate) in the past, during episodes of severe hyperkalemia, but it had been discontinued a few weeks before the ER visit due to an excessive reduction in potassium levels. No history of allergies was reported.

On clinical observation in the ER, the patient presented a deep ulcer with tendon exposure and perilesional swelling - cellulitis - in the right foot, associated with necrosis of the ipsilateral second and third toes. Abdomen examination was unremarkable. The patient had to be admitted for intravenous (IV) antibiotics and surgical debridement of the ulcer, with amputation of the second and third toes of the right foot. Despite long-term Piperacillin/tazobactam IV and local surgical intervention, the foot lesion continued to worsen and the patient had to endure amputation of the right leg on the 22^nd^ day of admittance.

After amputation, he developed hyperkalemia (K^+^ 6.0 mmol/L), which did not respond to dietary potassium restriction and insulin dose increase, and CPS was therefore prescribed. Potassium levels decreased steadily but more extensively than expected, and on the seventh day of treatment, resins were suspended. Despite this, his hypokalemia continued to worsen in the following days to levels as low as 2.0 mmol/L, and iv potassium supplementation was required in large amounts. Spironolactone was also prescribed. The patient complained of constipation and slight abdominal discomfort that could be solely attributed to hypokalemia, but was able to maintain a stool output every other day, so a major complication was unsuspected at this time. The consulting nephrologists suggested that a rare event - the development of a bezoar of ion-exchange resin - was a likely explanation for an unresponsive hypokalemia in this setting.

**Figure 1:**
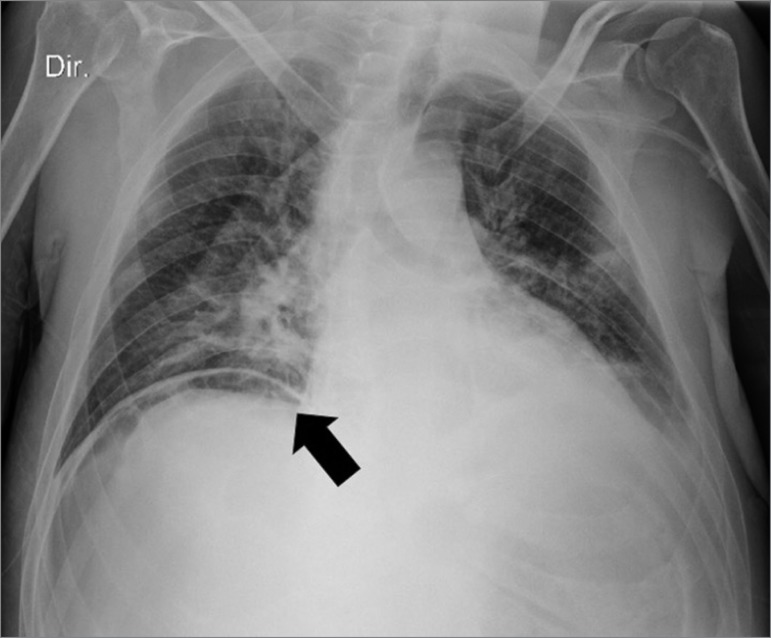
Patient’s thoracic radiography showing a pneumoperitoneum (black arrow).

On the 33^th^ day of hospitalization, the patient complained of diffuse abdominal pain and general weakness. His abdomen was distended and painful, with peritoneal reaction. The blood tests showed an elevation of infection parameters (leucocytes 10.6x10^3^µL with 89.6% neutrophil count; C-reactive protein of 25.6 mg/dL) that seemed to have no association with the initial clinical picture, as the amputation stump was clean with no signs of infection. Radiography revealed a pneumoperitoneum (Figure 1) and the patient was immediately transferred to the operation room for an exploratory laparotomy: a cecum perforation with peritonitis was diagnosed. This prompted an ileocecal resection with ileostomy and iv broad-spectrum antibiotics prescription (Imipenem/Cilastatin). During the surgical procedure, a solid white mass was removed from the lumen of the resected cecum, interpreted as a CPS bezoar.

**Figure 2:**
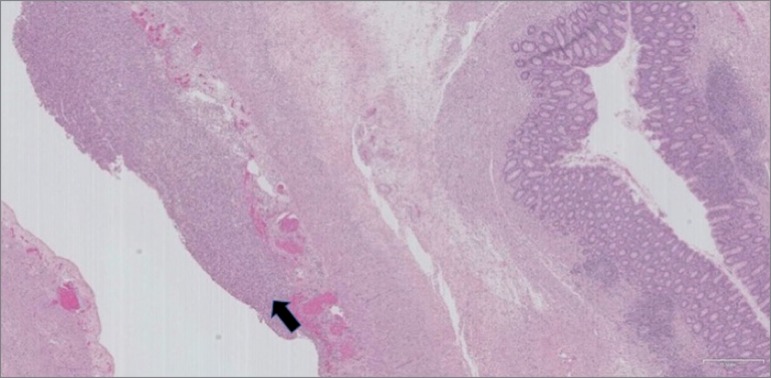
Histopathological findings of the resected fragment showing serositis (black arrow), and transmural ischemia.

The resected colon presented greyish external surface, hemorrhagic foci, and whitened plaques. Histologic examination (Figure 2) showed serositis and transmural ischemia. Whether cecum perforation was favored by mucosal ulceration from exposure to the resin or from lumen obstruction by the CPS bezoar was not completely established by the pathologist.

*Enterococcus faecium* was latter isolated in the peritoneal effusion and blood cultures. Unfortunately, despite every support measures taken in the Intensive Care Unit, the patient died one week after the colectomy due to septic shock.

## Discussion

PB are associated with many adverse events, mostly gastrointestinal, ranging from mild constipation to rare life-threatening complications such as the one described in this case report. Severe gastrointestinal adverse effects including colonic perforation have been documented in both type of resins, sodium and calcium polystyrene sulfonate, either with sorbitol or alone^4^. Although the colon is the most common location of injury, it is increasingly recognized that injury may occur in more proximal sections of the GIT: in 30% of the cases there is an injury in the esophagus, stomach, or small intestine^5^.

The pathophysiologic changes in the mucosa exposed to PB are usually mild but quite erratic, ranging from mucosal edema, ulceration, pseudomembranous colitis and transmural necrosis^6^. Haupt HM et al. have demonstrated that inoculation of tissue with SPS can lead to an inflammatory reaction within 24 hours and the release of cytokines and prostaglandins result in further impairment in local hemodynamic mechanisms, that culminate in vascular injury and mucosal lesion^7^. Ziv Harel et al. identified 58 cases of severe gastrointestinal adverse events associated with SPS use in a review of case series and case reports including frank necrosis and perforation^5^. Therefore, resins use alone can frequently induce GIT lesion, regardless of forming a drug bezoar and inducing bowel obstruction. In some cases, crystals of the drug can be found when assessing the pathologic sample, therefore corroborating the presence of the resin as the etiological agent of the lesion. However, despite existing *in vivo*, these crystals can often be lost during the physical preparation of the sample thus remaining undetectable.

In the present case, the onset of severe hypokalemia, despite discontinuation of CPS, enduring for days and requiring iv potassium supplementation, was highly suggestive of an unremitting PB influence, best explained by the sustained presence of the drug inside the GI tract. The best assumption was that a CPS bezoar had formed inside the intestinal lumen.

A bezoar is a stiff, solid, and persistent foreign body that is located in the GIT. The majority of bezoars are located in the stomach; however, they may be encountered in the whole GIT, including the esophagus and colon. Depending on the material of origin, four different types of bezoars have been described: phytobezoars (hortobezoars), trichobezoars (pilobezoars, hairball), stone-like foreign bodies, and pharmacobezoars (drug-induced)^8^. There is little knowledge on pharmacobezoars, as there are only nearly 30 published articles on the subject. The majority of pharmacobezoars develop in the stomach^8^, formed by the anomalous binding of drugs due to alterations in GIT anatomical structure, motility, or secretion. It is thus expected that the most common risk factor is a history of previous gastric surgery^9^. DM and antacid drug use are other recognized predisposing factors. The clinical diagnosis is usually difficult; therefore, pharmacobezoars are usually diagnosed during an operation or endoscopy^8^.

Concerning PB use, numerous risk factors have been identified as contributors to the gastrointestinal injury induced by these drugs: CKD and ESRD (elevated renin levels predispose patients to non-occlusive mesenteric ischemia through angiotensin II-mediated vasoconstriction); postoperative status (hypotension, ileus-induced colonic distension with consequent reduced colonic blood flow, and decreased gut motility as a result of opioids; constipation increases the risk of injury); and solid organ transplantation (there might be an increased risk associated with immunosuppressive medications that impair normal protective and reparative mechanisms of gastrointestinal cells)^5^. PB should be avoided in these patients, or at least prescribed in a small dosage, with frequent monitoring. In view of the present case of a PB bezoar development in a patient with history of partial gastrectomy and vagothomy, the authors believe that there should be a warning for PB prescription in such patients.

There is no specific treatment guidelines for PB bezoars, only for their complications. Neither endoscopy nor laparotomy are advocated in an early stage to remove the resin mass. The use of osmotic cathartics should also be avoided. The current recommendation is drug interruption, along with hemodynamic improvement to prevent gastrointestinal hypoperfusion that could lead to transmural necrosis, which was the conduct taken in this case^10^. Unfortunately, cecum perforation occurred a few days later. In the setting of free perforation to abdomino-pelvic cavity, the surgeon must seek the removal of PB crystals from the peritoneum. On-table colonic lavage can be used to make sure that all the resin is removed from the lumen and the creation of a primary anastomosis is ill-advised^10^. There is frequent need for surgical re-exploration due to new intestinal perforations that can occur from PB intraluminal or peritoneal remnants^10^.

The future of acute and chronic hyperkalemia treatment is likely to be altered by two emerging and promising therapies: patiromer and sodium zirconium cyclosilicate (Table 2). Patiromer (Veltassa^®^), approved by the Food and Drug Administration (FDA) in October 2015 and by the European Medicines Agency (EMA) in July 2017, is a non-absorbable organic polymer that binds potassium in exchange for calcium, mostly in distal colon, where the concentration of free potassium is highest^3^. It is a non-selective cation-exchanger, with action onset around 7 hours and effects lasting through 48 hours^11^. There are cases of hypomagnesemia and constipation associated with patiromer, however it seems to be better tolerated when compared to other available PBs^3^.

**Table 2:** Comparison between the two new hyperkalemia therapies recently introduced in the market

	POLYSTYRENE SULFONATE (PS)	PATIROMER	ZS-9: SODIUM ZIRCONIUM CYCLOSILICATE
STRUCTURE	Sulphonated cross-linked polystyrene copolymer.	Spherical organic polymer (oral suspension).	Microporous crystalline spherical inorganic polymer (powder).
MECHANISM	Not absorbed. Exchanges K^+^ for Ca^2+^ or Na^+^. Non-selective binding.	Not absorbed. Exchanges K^+^ for Ca2^+^. Non-selective binding.	Not absorbed. Exchanges K^+^ for Na^+^. Highly selective for K^+^ (125x superior than PS).
ACTION	Stomach and mainly colon.	Distal colon. Sustained effects for 24-48h.	Duodenum, jejunum, Ileum and colon.
CKD POPULATION	Tested in CKD population.	Tested in CKD population.	Tested in CKD population.
MAIN ADVERSE EVENTS	Abdominal pain. Diarrhea or constipation. Intestinal ulceration/perforation. Hypercalcemia, hypokalemia.	Hypomagnesemia. Hypokalemia. Constipation.	Edema. Hypokalemia.
MAIN ADVANTAGES	Potassium binding.	Better tolerated. Calcemia control in CKD. Compatible with RAASi.	Better tolerated. Powerful K+ binding effect. Compatible with RAASi.

Sodium zirconium cyclosilicate, also known as ZS-9 (Lokelma^®^), is a 2018 FDA/EMA approved highly selective inorganic microporous cation exchanger that entraps potassium in the intestinal tract in exchange for sodium and hydrogen. Its great advantage is that it has 9.3 times more potassium-binding capacity than SPS and is more than 125 times more selective for potassium than the former. Although some GI adverse events have been described associated with ZS-9, such as edema and hypokalemia, several studies have shown that the drug has good safety profile, capable of consistently reducing serum potassium levels^3^.

In conclusion, CPS and SPS administration can lead to severe gastrointestinal adverse events. Lack of an alternative drug for the treatment of chronic hyperkalemia makes the use of these drugs common and render their side effects rather frequent. The authors describe a rare case of a PB pharmacobezoar, seldom diagnosed, that contributed to cecum perforation. They believe that the partial gastrectomy and vagothomy (performed several years before for treatment of duodenal ulcer with stenosis) was an important risk factor for PB bezoar development, and suggest that in such patients an alternative treatment option for hyperkalemia should be sought. The recent development of Patiromer and ZS-9 as new PBs might change the paradigm of hyperkalemia therapy.
